# Association between morphologic features of intracranial distal arteries and brain atrophy indexes in cerebral small vessel disease: a voxel-based morphometry study

**DOI:** 10.3389/fneur.2023.1198402

**Published:** 2023-06-15

**Authors:** Hongjiang Cheng, Junfang Teng, Longbin Jia, Lina Xu, Fengbing Yang, Huimin Li, Chen Ling, Wei Liu, Jinna Li, Yujuan Li, Zixuan Guo, Xia Geng, Jiaying Guo, Dandan Zhang

**Affiliations:** ^1^Department of Neurology, The First Affiliated Hospital of Zhengzhou University, Zhengzhou, Henan, China; ^2^Department of Neurology, Jincheng People’s Hospital, Jincheng, Shanxi, China; ^3^Graduate School, Changzhi Medical College, Changzhi, Shanxi, China

**Keywords:** cerebral small vessel disease, brain atrophy, intracranial distal arteries, morphologic features, voxel-based morphometry

## Abstract

**Background:**

Brain atrophy represents a final common pathway for pathological processes in patients with cerebral small vessel disease (CSVD) and is now recognized as a strong independent predictor of clinical status and progression. The mechanism underlying brain atrophy in patients with CSVD is not yet fully comprehended. This study aims to investigate the association of morphologic features of intracranial distal arteries (A2, M2, P2 and more distal) with different brain structures [gray matter volume (GMV), white matter volume (WMV), and cerebrospinal fluid volume (CSFV)]. Furthermore, we also examined whether a correlation existed between these cerebrovascular characteristics and GMV in different brain regions.

**Method:**

A total of 39 participants were eventually enrolled. The morphologic features of intracranial distal arteries based on TOF-MRA were extracted and quantified using the intracranial artery feature extraction technique (iCafe). The brain 3D-T1 images were segmented into gray matter (GM), white matter (WM), and cerebrospinal fluid (CSF) using the “Segment” tool in CAT12 for the voxel-based morphometry (VBM) analysis. Univariable and multivariable linear regression models were used to investigate the relationship between these cerebrovascular features and different brain structures. Partial correlation analysis with a one-tailed method was used to evaluate the relationship between these cerebrovascular features and GMV in different brain regions.

**Results:**

Our findings indicate that both distal artery length and density were positively correlated with GM fraction in CSVD patients, regardless of whether univariable or multivariable linear regression analyses were performed. In addition, distal artery length (*β* = −0.428, *p* = 0.007) and density (*β* = −0.337, *p* = 0.036) were also found to be negative associated with CSF fraction, although this relationship disappeared after adjusting for potential confounders. Additional adjustment for the effect of WMHs volume did not change these results. In subgroup anasysis, we found that participants in the highest distal artery length tertile had significantly higher GM fraction and lower CSF fraction level than participants in the lowest distal artery length tertile. In partial correlation analysis, we also found that these cerebrovascular characteristics associated with regional GMV, especially subcortical nuclear.

**Conclusion:**

The morphologic features of intracranial distal arteries, including artery length, density and average tortuosity, measured from 3D-TOF MRA, are associated with generalized or focal atrophy indexes of CSVD.

## Introduction

The neuroimaging features of cerebral small vessel disease (CSVD) comprise a range of abnormalties, such as small subcortical infarcts, lacunes, white matter hyperintensities (WMHs), enlarged perivascular spaces, cerebral microbleeds (CMBs), and brain atrophy (1). Brain atrophy represents a final common pathway for pathological processes and is now recognized as a strong independent predictor of clinical status and progression in patients with CSVD (2–4). The impact of vascular lesions associated with CSVD on clinical status is, to some extent, influenced by alterations in brain atrophy and cortical morphology (5, 6). Brain volume, which is another imaging indicator of brain atrophy, has been found to be associated with cognition, gait abnormality and disability scales (7–9). In addition, in statistical models, brain volume regularly outperform other lesion-based magnetic resonance imaging (MRI) markers of CSVD for predicting cognitive outcomes (10). Structural MRI can provide morphological information that reveals the cortical and subcortical deformation in response to pathological changes. Voxel-based morphometry (VBM) is a well proven neuroimaging analysis technique that enables investigation of focal differences in brain volume, using the statistical approach of statistical parametric mapping (SPM) (11).

The mechanism underlying brain atrophy in patients with CSVD is not yet fully comprehended. Neuropathological hallmarks of atrophy include neuronal loss, cortical thinning, subcortical vascular pathology with white matter rarefaction and shrinkage, arteriolosclerosis, venous collagenosis, and secondary neurodegenerative changes (12–14). There is no direct pathological study avaliable to elucidate the histological foundation of brain volume changes in patients with CSVD. Brain atrophy may be a direct result of microvascular lesions. Furthermore, brain atrophy may also be secondary to other processes, such as white matter tract disruption with secondary atrophy and focal cortical thinning in brain regions connected to subcortical infarcts (3, 15, 16). Therefore, evaluating the association between vascular injury and brain atrophy in CSVD holds significant value for further mechanism study. A major hurdle is that the microvascular lesions involved are too minute to be directly and quantitatively measured. Therefore, discovering an alternative quantitative marker of cerebrovascular injury is of immense importance.

Three dimensional time-of-flight (3D-TOF) magnetic resonance angiography (MRA) is the most common angiography technology in clinical practice, extensively employed for the evaluation of head and neck arteries (17). The advancement of image processing technology has enabled the extraction of cerebral vascular features based on 3D-TOF MRA. It differs from the blood flow information of the proximal arteries or brain parenchyma, as it mainly focuses on the distal arteries (A2, M2, P2 and more distal). Recently developed and validated, the intracranial artery feature extraction technique (iCafe) can effectively evaluate the morphometry and intensity features of all vascular regions identified in the brain, based on 3D-TOF MRA images (18). The reproducibility analysis of iCafe demonstrated outstanding agreement for both inter-scan and intra-operator evaluations, and good agreement for inter-operator evaluations, indicating the quantitative measurements of intracranial arteries are highly reliable (19). Furthermore, Gould et al. (20) discovered that the vascular features of distal arteries on 3D-TOF MRA, such as vessel length and the number of branches, may serve as surrogate markers of blood flow. Moreover, these vascular features may also be affected by other factors, such as wall thickening-induced luminal narrowing in the distal arteries (21). Therefore, these vascular features may provide distinctive information regarding blood flow and luminal narrowing in distal arteries. Chronic hypertension is one of the most important risk factors for CSVD (22). Studies on essential hypertension suggest that structural alterations of small and large vessels are closely interdependent (23–26), implying that the hypothesis that small vessel pathological changes may be associated with those in large vessels. These studies provide a theoretical basis for the hypothesis that the morphologic features of intracranial distal arteries are associated with brain atrophy in CSVD.

In this study, we aimed to use iCafe to quantify the morphologic features of intracranial distal arteries, including artery length, density and average tortuosity, measured from 3D-TOF MRA and investigate their relationship with different brain structures [gray matter volume (GMV), white matter volume (WMV), and cerebrospinal fluid volume (CSFV)]. Furthermore, we also examined whether a correlation existed between these cerebrovascular characteristics and GMV in different brain regions. Our study updated the method for evaluating the morphological characteristics of intracranial arteries in CSVD, providing valuable information to clarify the relationship between vascular health indicators and brain atrophy, and offering new evidence for a possible vascular origin of brain atrophy.

## Materials and methods

### Participants

**Figure 1:**
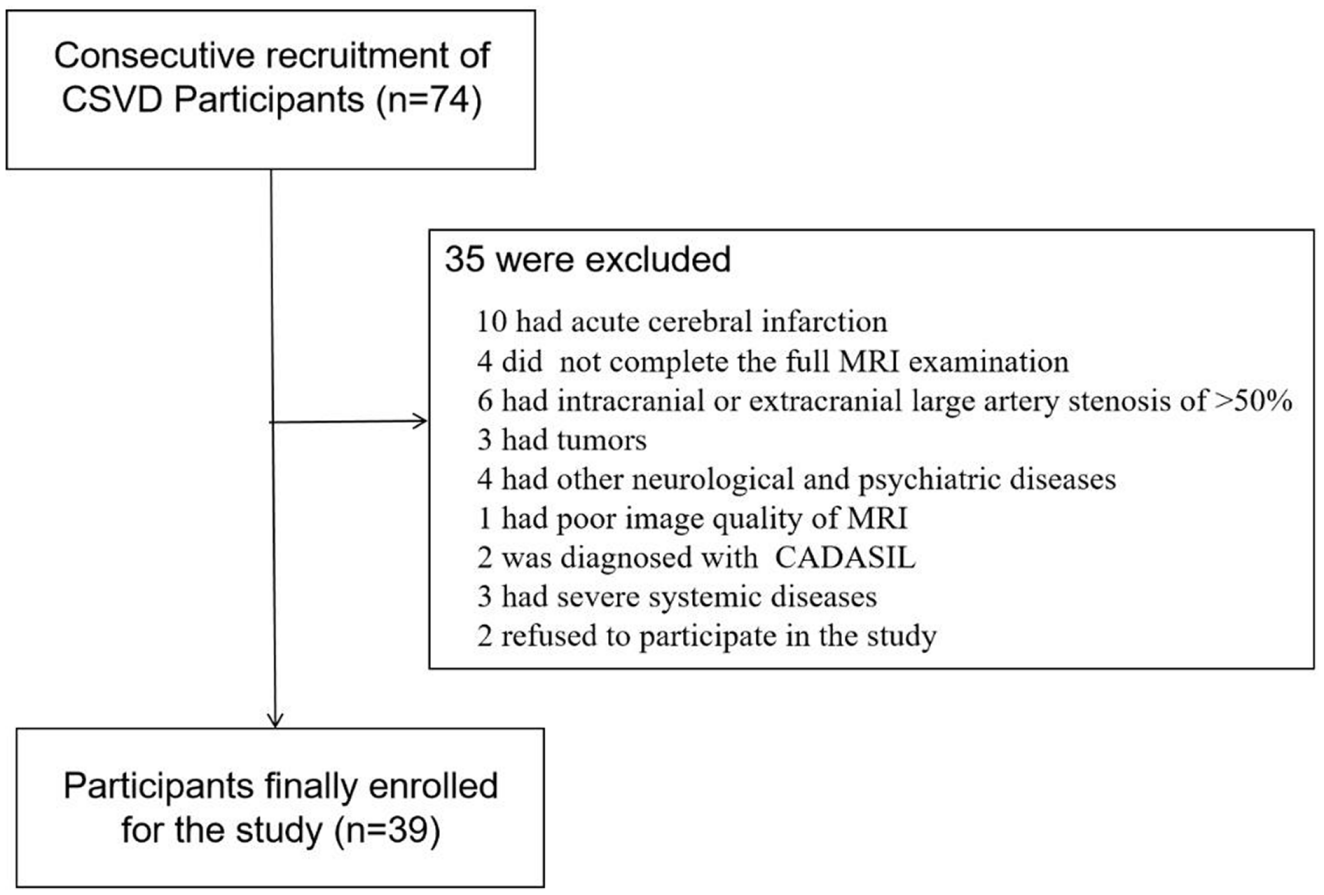
Flowchart of screened, excluded, and included cerebral small vessel disease (CSVD) participants among the outpatients and inpatients in the Department of Neurology between June 2022 to December 2022.

Seventy-four participants diagnosed with CSVD were consecutively recruited from the outpatients and inpatients of the Department of Neurology between June 2022 and December 2022. Following the inclusion and exclusion criteria, we enrolled thirty-nine participants in the study. The specific screening process is illustrated in Figure 1. Ethical approval was obtained from the ethics committee of Jincheng People’s Hospital and written informed consent was obtained from all participants. WMHs was defined as hyperintense on T2-weighted or FLAIR sequences and can appear as isointense or hypointense (although often not as hypointense as CSF) on T1-weighted sequences (1). WMHs were graded using the Modified Fazekas Scale and recorded as 0 = no, 1 = punctate, 2 = beginning, and 3 = confluent (27). Participants with WMHs of a Modified Fazekas scale≥2 were considered as having CSVD, with or without lacunar infarction (LI), perivascular spaces and microbleeds. Demographic data and vascular risk factors, including age, gender, body mass index (BMI), hypertension, diabetes mellitus, heart disease, history of smoking and past history of stroke or transient ischemic attack (TIA) were recorded. Metabolic markers including cholesterol, low-density lipoproteins, glycosylated hemoglobin and homocysteine levels were also collected.

The inclusion criteria for this study were as follows: (a) age between 50–80 years; (b) meeting the diagnostic criteria of CSVD (as described above) (1); and (c) all participants had to sign the informed consent form prior to study entry. The exclusion criteria were as follows: (a) acute cerebral infarction in recent 3 months; (b) cerebral infarctions>20 mm in diameter; (c) leukoencephalopathy of non-vascular origin (e.g., immunological demyelination, metabolic, toxic or infectious diseases); (d) intracranial or extracranial large artery stenosis of >50%; (e) intracranial hemorrhage; (f) history of hydrocephalus, cerebral tumor or space occupying lesions; (g) hereditary CSVD; (h) severe systemic disease; (i) other neurological and psychiatric diseases, such as Alzheimer’s Disease (AD), Parkinson’s disease (PD), neurological infection, epilepsy, schizophrenia and bipolar disorder; (j) formal contraindications or refusal to undergo cerebral MRI and (k) left-handedness.

### MRI protocol and image processing

All participants underwent MRI using a standard protocol. Scans were obtained using a 3.0T Philips Intera scanner (Ingenia 3.0T, Philips Medical Systems, The Netherlands) at the Imaging Department of Jincheng People’s Hospital. The protocol included the following sequences: T1-weighted [repetition time (TR) = 1800 ms, echo time (TE) = 20 ms, flip angle (FA) = 90°, slices = 20, the field of view (FOV) = 230 × 185 mm^2^, acquisition matrix =328 × 199, thickness = 5.5 mm, voxel size =0.7 × 0.88 × 5.5 mm^3^]，T2-weighted (TR = 4,000 ms, TE = 107 ms, FA = 90°, slices = 20, FOV = 230 × 230 mm^2^, acquisition matrix = 384 × 384, thickness = 5 mm, voxel size = 0.6 × 0.6 × 5 mm^3^), FLAIR (TR = 11,000 ms, TE = 120 ms, FA = 90°, slices = 20, FOV = 230 × 187 mm^2^, acquisition matrix = 356 × 115, thickness = 5.5 mm, voxel size = 0.65 × 1 × 5.5 mm^3^), TOF-MRA (TR = 23 ms, TE = 3.5 ms, FA = 18°, slices = 140, FOV = 200 × 200 mm^2^, acquisition matrix = 444 × 294, thickness = 1.2 mm, voxel size = 0.45 × 0.68 × 1.2 mm^3^), 3D-T1(TR =7.9 ms, TE = 3.5 ms, FA = 8°, slices = 360, FOV = 256 × 256 mm^2^, acquisition matrix = 256 × 256, thickness = 1 mm, voxel size =1 × 1 × 1 mm^3^).

Image preprocessing and VBM analysis were performed using SPM12 program^1^ on the MATLAB R2021b platform. The image preprocessing steps performed were based on a standard protocol.^2^ Using the “Segment” tool in CAT12, the brain 3D-T1 images of every participant were skull-stripped and segmented into white matter (WM), grey matter (GM) and cerebrospinal fluid (CSF), which were used to determine regional GM, WM and CSF volumes, and to calculate total intracranial volume (TIV = GM + WM + CSF). Segmented GM maps were warped to the standard diffeomorphic anatomical registration through exponentiated lie (DARTEL) algebra template and normalized to the Montreal Neurological Institute (MNI) space (28). Normalized GM maps were then modulated to obtain the volume of GM tissue corrected for individual brain sizes and smoothed with an 8-mm full width at half maximum isotropic Gaussian kernel. Finally, the GM of the whole brain was divided into 170 regions using the automated anatomical labelling 3(AAL3) atlas (29).

**Figure 2:**
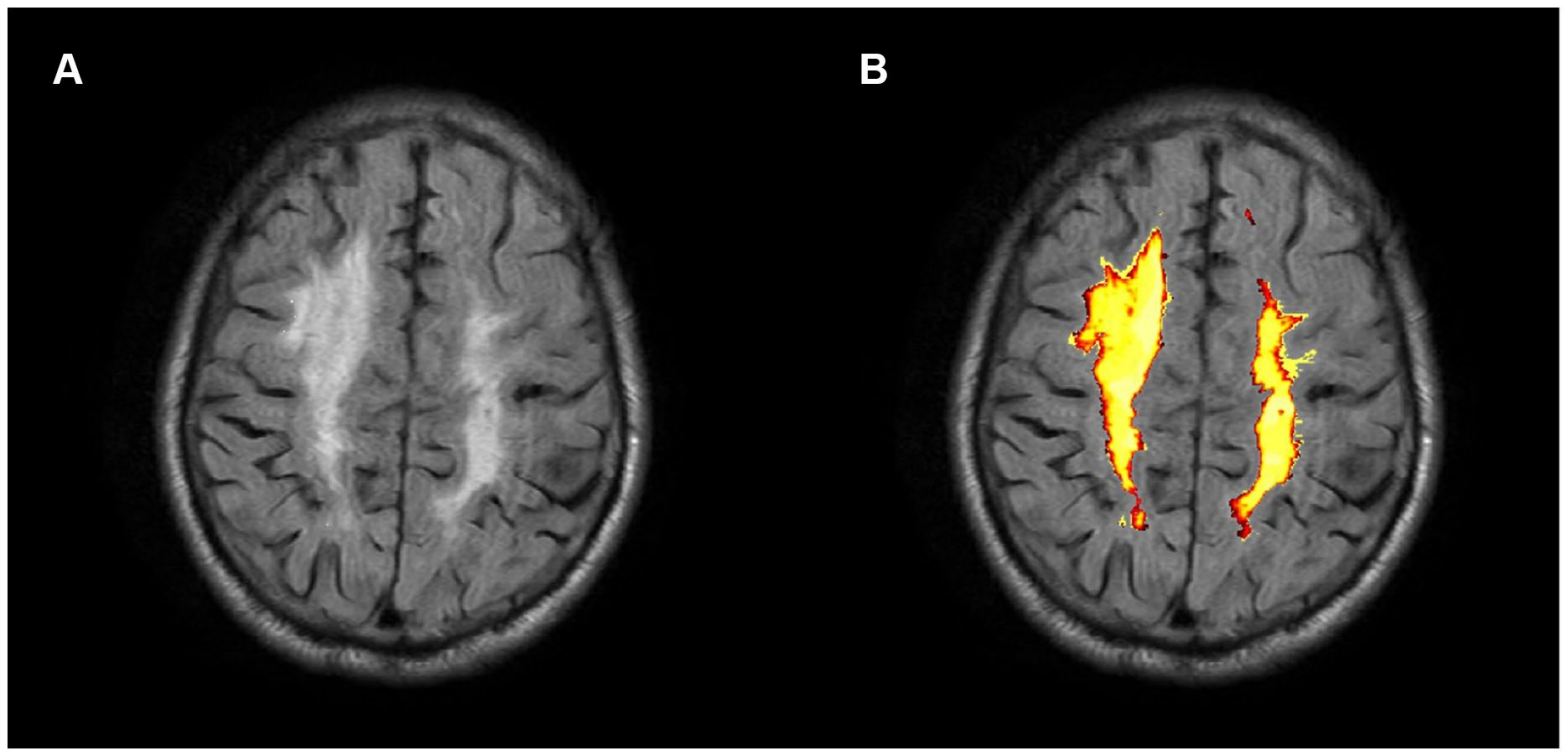
Axial FLAIR image of a CSVD participant **(A)** and associated probabilistic lesion volume map **(B)** generated by the LST-LPA.

WMHs volumes were automatically estimated using the lesion prediction algorithm (LPA) provided by the lesion segmentation tool (LST) toolbox (30)^3^ for SPM 12 (Figure 2). The detection of WMHs lesions only utilized FLAIR images.

**Figure 3:**
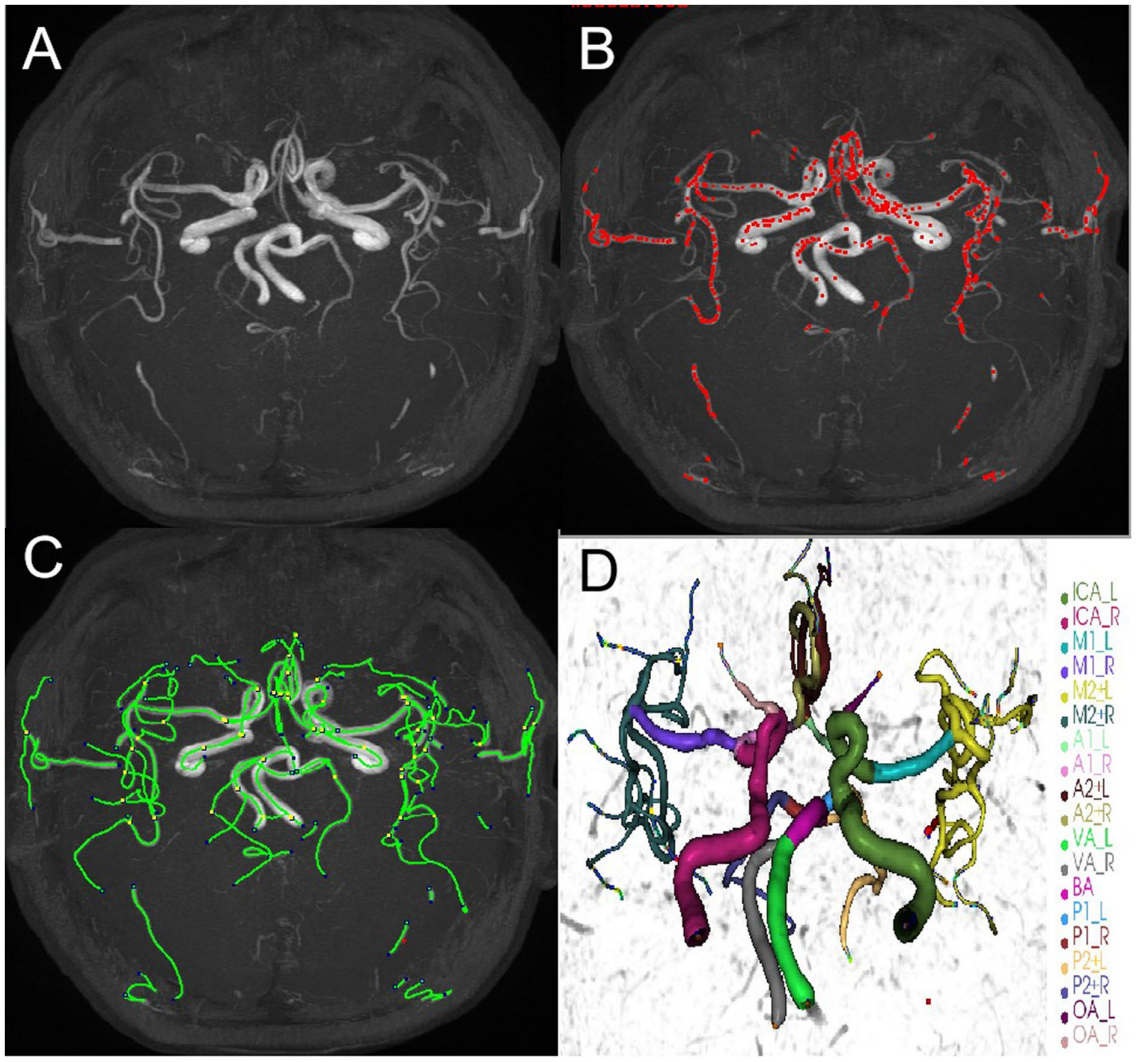
An example of iCafe processed 3D-TOF MRA of CSVD participant in maximum intensity projection (MIP) **(A–D)**. The different intracranial arteries are indicated with different colors in the vessel tracing results **(D)**.

All the brain MRA images were processed using iCafe. Arteries in 3D-TOF MRA were traced using an open-curve active contour model (31) and reconstructed as radius-varying tubes. To address the artery tracing problems caused by artery boundary blurring due to pulsation and global image quality deterioration due to the motion during MRA scan, an artery trace refinement algorithm was used to correct centerline deviations and erroneous radius estimations along the traces (32). Subsequently arteries were labeled as one of the 24 anatomical types, allowing for comprehensive regional-based arterial features to be extracted, such as artery length, volume and tortuosity of intracranial distal arteries (A2, M2, P2 and more distal) (18, 33). The process was illustrated in Figure 3. The distal artery group consisted of the M2 and M3+ segments of the MCA, the A2+ segments of the ACA and the P2+ segments of the PCA. Artery length refers to the end-to-end distance of the centerline of an artery. Density is defined as the ratio of arterial volume and brain parenchymal volume. Tortuosity is the ratio between artery length and the Euclidean distance of the 2 terminal points of an individual artery segment. Cheng and Li, who have extensive experience in neuroimaging analysis, conducted these analyses above.

### Statistical analyses

Continuous variables with normal distribution were presented as mean ± standard deviation (SD). Variables with non-normal distribution were presented as median (interquartile ranges), while categorical variables were presented as frequencies (percentages). The normality of continuous variables was assessed by the Shapiro–Wilk test.

To examine the associations between morphologic features of intracranial distal arteries (artery length, density, and average tortuosity) and different brain structures (GMV, WMV, and CSFV), univariable linear regression was performed with different brain structures as the dependent variable and these cerebrovascular characteristics as the independent variable. Subsequently, multivariable linear regressions that include potential confounders were performed. To identify other confounders, univariable linear regression between each clinical variable and different brain structures was performed, and only the clinical variables with a *p* < 0.1 were chosen as confounders. To mitigate the influence of brain size, the distal artery length was normalized with the cube root of total brain volume (TBV), while the WMHs volume was normalized with TBV.

Furthermore, the participants were stratified into three groups according to the tertile of the distal artery length: 1st tertile, 2nd tertile and 3rd tertile. Subgroup analysis was performed to compare clinical characteristics and imaging features between participants in the two extreme tertiles (i.e., lowest and highest tertiles) of distal artery length distribution. Categorical variables were compared using Fishers exact tests, while continuous variables were compared using *t*-tests or Wilcoxon rank-sum tests, as appropriate.

Partial correlation analysis with a one-tailed approach was employed to evaluate the relationship between the morphologic features of intracranial distal arteries and GMV in different brain regions. Age, gender and total intracranial volume (TIV) were regarded as covariates in partial correlation analysis. The false discovery rate (FDR) statistics were calculated for each *p*-value, and those with adjusted *p*-values of *<*0.05 were regarded significant.

## Results

### Baseline characteristics

A total of 39 participants, aged between 50 to 80 years old, with a mean age of 68.08 years (SD = 6.56), were enrolled in the study and all participants successfully completed the MR imaging. Clinical data were collected upon admission, and laboratory data were collected within 24 h of admission. The data are tabulated in Table 1.

**Table 1:** Baseline characteristics of the participants.

Baseline characteristics	CSVD (*n* = 39)
Age (years)	68.08 (6.56)
Male sex, No. (%)	28 (71.8)
BMI	23.51 (4.59)
**Medical history**	
Hypertension	28 (71.8)
Diabetes	11 (28.2)
Coronary artery disease	9 (23.1)
Smoking history	8 (20.5)
History of stroke or TIA	19 (48.7)
**Laboratory findings**	
Total cholesterol, mmol/L	3.5 (0.94)
Homocysteine, μmol/L	17.4 (20.35)
Low-density lipoproteins, mmol/L	2.08 (0.85)
Glycosylated hemoglobin (%)	5.8 (0.78)
**Imaging features**	
Normalized WMHs volumes (10^−2^)	3.29 (2.95)
GM fraction (%)	39.56 (3.07)
WM fraction (%)	34.3 (5.01)
CSF fraction (%)	26.9 (4.53)

Values represented as mean (SD), number (%), or median (IQR). BMI, body mass index; TIA, transient ischemic attack.

### Association between the morphologic features of intracranial distal arteries and different brain structures

Our study analyzed brain structures using the GM fraction (calculated by dividing GMV by TIV), WM fraction (calculated by dividing WMV by TIV), and CSF fraction (calculated by dividing CSFV by TIV). Distal vascular features were evaluated using artery length, density, and average tortuosity. Tables 2–4 present the results of univariable and multivariable linear regression analyses investigating the associations between intracranial distal vascular features and different brain structures. The results of the univariate linear regression model showed that distal artery length (*β* = 0.535, *p* < 0.001) (Table 2) and density (*β* = 0.507, *p* = 0.001) (Table 3) had a significant correlation with GM fraction. Furthermore, age (*p* = 0.022) and history of stroke or TIA (*p* = 0.045) exhibited *p*-values of less than 0.1 in the univariable linear regression analysis for association with GM fraction and were thus included as confounding factors in the multivariable linear regression model. After adjusting for these clinical confounding factors, the associations between distal artery length (*β* = 0.418, *p* = 0.007) (model 1 in Table 2) and density (*β* = 0.431, *p* = 0.005) (model 1 in Table 3) and GM fraction remained statistically significant. Additional adjustment for the effect of WMHs volume did not change these results (model 2 in Tables 2, 3). The univariate linear regression model also revealed correlations between distal artery length (*β* = −0.428, *p* = 0.007) (Table 2) and density (*β* = −0.337, *p* = 0.036) (Table 3) and CSF fraction. However, these associations disappeared after adjusting for age (*p* = 0.002), smoking history (*p* = 0.023), and history of stroke or TIA (*p* = 0.007). No significant associations were found between distal artery length (Table 2) and density (Table 3) and WM fraction in either the univariable or multivariable regression analysis. Furthermore, no significant correlations between average tortuosity and different brain structures were found in the statistical analysis (Table 4).

**Table 2:** Univariable and multivariable linear regression analyses of distal artery length and brain structures (*N* = 39).

Brain structure	Univariable linear regression			Multivariable linear regression					
				Model 1			Model 2		
	*β*	*p*	Adjusted *R*^2^	*β*	*p*	Adjusted *R*^2^	*β*	*p*	Adjusted *R*^2^
GM fraction	0.535	**<0.001**	0.267	0.418	**0.007**	0.311	0.418	**0.008**	0.3
WM fraction	0.099	0.549	−0.017	−0.105	0.524	0.149	−0.103	0.534	0.139
CSF fraction	−0.428	**0.007**	0.1613	−0.205	0.145	0.397	−0.207	0.141	0.398

GM, gray matter; WM, white matter; CSF, cerebrospinal fluid. Model 1 was adjusted for clinical confounding factors; model 2 was model 1 plus adjustment for WMHs volume. Bold values: significant *p*-values (*p* < 0.05).

**Table 3:** Univariable and multivariable linear regression analyses of distal artery density and brain structures (*N* = 39).

Brain structure	Univariable linear regression			Multivariable linear regression					
				Model 1			Model 2		
	*β*	*p*	Adjusted *R*^2^	*β*	*p*	Adjusted *R*^2^	*β*	*p*	Adjusted *R*^2^
GM fraction	0.507	**0.001**	0.237	0.431	**0.005**	0.325	0.426	**0.006**	0.307
WM fraction	0.008	0.959	−0.027	−0.137	0.4	0.157	−0.163	0.326	0.155
CSF fraction	−0.337	**0.036**	0.090	−0.217	0.118	0.402	−0.2	0.157	0.394

GM, gray matter; WM, white matter; CSF, cerebrospinal fluid. Model 1 was adjusted for clinical confounding factors; model 2 was model 1 plus adjustment for WMHs volume. Bold values: significant *p*-values (*p* < 0.05).

**Table 4:** Univariable and multivariable linear regression analyses of average tortuosity and brain structures (*N* = 39).

Brain structure	Univariable linear regression			Multivariable linear regression					
				Model 1			Model 2		
	*β*	*p*	Adjusted *R*^2^	*β*	*p*	Adjusted *R*^2^	*β*	*p*	Adjusted *R*^2^
GM fraction	0.169	0.304	0.002	0.135	0.369	0.169	0.12	0.444	0.149
WM fraction	−0.033	0.842	−0.026	−0.043	0.782	0.141	−0.083	0.606	0.136
CSF fraction	−0.092	0.577	−0.018	−0.082	0.539	0.364	−0.049	0.722	0.358

GM, gray matter; WM, white matter; CSF, cerebrospinal fluid. Model 1 was adjusted for clinical confounding factors; model 2 was model 1 plus adjustment for WMHs volume.

### Subgroup analysis between participants at extreme Tertile of the distal artery length levels

The subgroup analysis comparing different brain structures between the extreme tertile of the distal artery length levels revealed that participants in the highest distal artery length tertile had significantly higher GM fraction level and lower CSF fraction than participants in the lowest distal artery length levels (Table 5).

**Table 5:** Subgroup analysis between participants in extreme tertile of the distal artery length and different brain structures.

Baseline characteristics	1st tertile group	3rd tertile group	*p*
Number	13	13	
Age (years)	68.69 (7.24)	66 (4.51)	0.156
Male sex, No. (%)	10 (76.9)	7 (53.8)	0.411
BMI	21.22 (8.41)	23.6 (2.37)	0.920
**Medical history**			
Hypertension, No. (%)	10 (76.9)	10 (76.9)	1
Diabetes, No. (%)	5 (38.5)	2 (15.4)	0.378
Coronary artery disease, No. (%)	2 (15.4)	2 (15.4)	1
Smoking history, No. (%)	2 (15.4)	4 (30.8)	0.645
History of stroke or TIA, No. (%)	8 (61.5)	4 (30.8)	0.238
**Laboratory findings**			
Total cholesterol, mmol/L	3.29 (1.16)	3.85 (1.02)	0.264
Homocysteine, μmol/L	30.66 (14.37)	14.2 (11.95)	**0.019**
low-density lipoproteins, mmol/L	2.09 (0.57)	2.47 (0.83)	0.186
Glycosylated hemoglobin (%)	5.9 (0.68)	5.8 (0.88)	0.579
**Imaging features**			
Normalized WMHs volumes (10^−2^)	3.62 (1.62)	3.13 (1.55)	0.448
GM fraction (%)	37.69 (3.4)	40.98 (1.96)	**0.006**
WM fraction (%)	33.57 (3.64)	34.02 (2.84)	0.727
CSF fraction (%)	28.74 (5.25)	25 (2.97)	**0.035**

Bold values: significant *p*-values (*p* < 0.05).

### Association between the morphologic features of intracranial distal arteries and GMV in different brain regions

**Figure 4:**
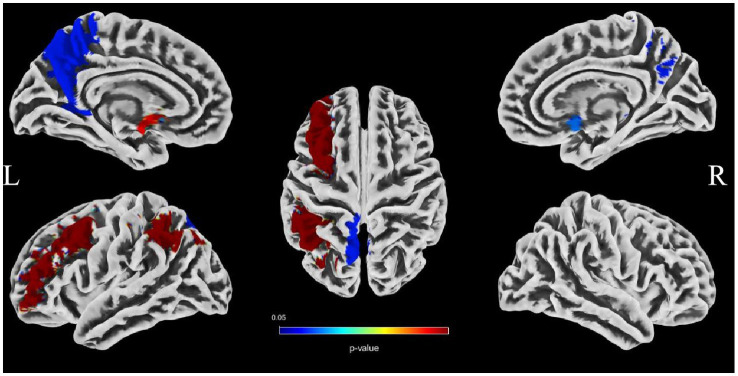
Images showing GM regions where distal artery length is positively associated with GMV (*p* < 0.05, FDR correction).

**Figure 5:**
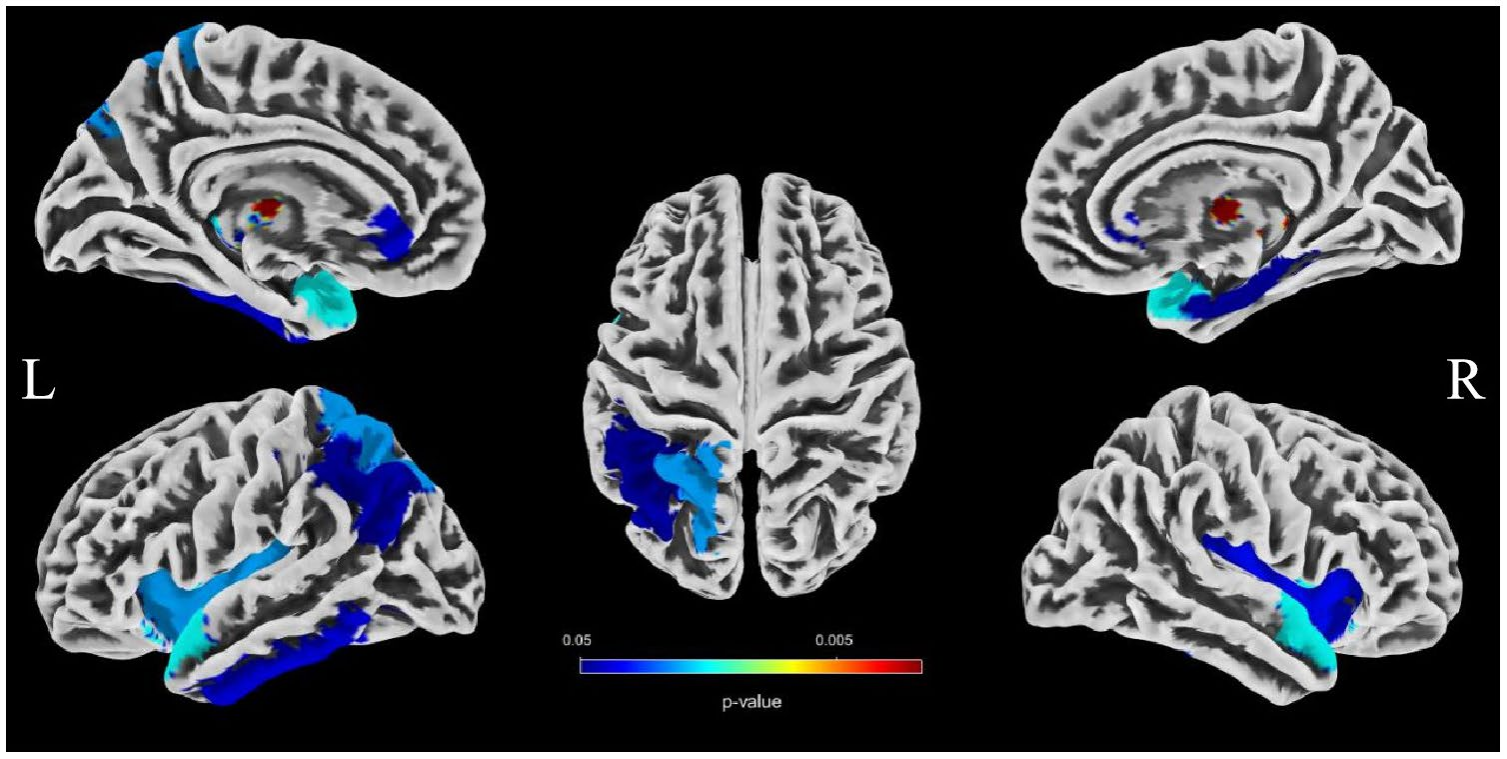
Images showing regions where average tortuosity of distal arteries is positively associated with GMV (*p* < 0.05, FDR correction).

To evaluate the associations between intracranial distal vascular features and GMV in different brain regions, partial correlation analysis with a one-tailed method was performed, controlling for gender, age and TIV. The AAL3 atlas was used to label statistically significant areas in the result reporting. The correlation analysis results are shown in Tables 6, 7, and the corresponding GM regions images are shown in Figures 4, 5. However, we did not find distal artery density related GM regions at our statistical cut off of FDR <0.05. No brain regions correlated negatively with these cerebrovascular characteristics.

**Table 6:** GM regions significantly positively correlated with distal artery length in patients with CSVD after adjusting for gender, age and TIV (*p* < 0.05, FDR correction).

Hemisphere	Region	Full names	*R*	*p-*value
Left	CAU	Caudate nucleus	0.616	0.005
	PUT	Lenticular nucleus, putamen	0.560	0.016
	IPG	Inferior parietal gyrus, excluding supramarginal and angular gyri	0.540	0.018
	MFG	Middle frontal gyrus	0.524	0.018
	CERCRU2	Crus II of cerebellar hemisphere	0.523	0.018
	Nacc	Nucleus accumbens	0.512	0.020
	PCUN	Precuneus	0.463	0.043
Right	Nacc	Nucleus accumbens	0.478	0.039
	tPuA	Thalamus, pulvinar anterior	0.467	0.043

**Table 7:** GM regions significantly positively correlated with average tortuosity of distal arteries in patients with CSVD after adjusting for gender, age, and TIV (*p* < 0.05, FDR correction).

Hemisphere	Region	Full names	*R*	*p-*value
Left	tMDm	Thalamus, mediodorsal medial magnocellular	0.600	0.002
	tMDl	Thalamus, mediodorsal lateral parvocellular	0.569	0.005
	tVL	Thalamus, ventral lateral	0.561	0.005
	CER7b	Lobule VIIB of cerebellar hemisphere	0.524	0.011
	tPuM	Thalamus, pulvinar medial	0.505	0.015
	tPuL	Thalamus, pulvinar lateral	0.502	0.015
	TPOsup	Temporal pole: superior temporal gyrus	0.495	0.015
	CERCRU2	Crus II of cerebellar hemisphere	0.475	0.019
	INS	Insula	0.470	0.021
	tPuA	Thalamus, pulvinar anterior	0.467	0.021
	SPG	Superior parietal gyrus	0.460	0.021
	CER8	Lobule VIII of cerebellar hemisphere	0.459	0.021
	tLGN	Thalamus, lateral geniculate	0.440	0.028
	tVPL	Thalamus, ventral posterolateral	0.422	0.033
	tLP	Thalamus, lateral posterior	0.421	0.033
	ITG	Inferior temporal gyrus	0.412	0.037
	tIL	Thalamus, intralaminar	0.410	0.037
	ACCsub	Anterior cingulate cortex, subgenual	0.409	0.037
	tVA	Thalamus, ventral anterior	0.395	0.042
	ANG	Angular gyrus	0.392	0.043
	tAV	Thalamus, anteroventral nucleus	0.384	0.046
	IPG	Inferior parietal gyrus, excluding supramarginal and angular gyri	0.383	0.046
Right	tMDm	Thalamus, mediodorsal medial magnocellular	0.628	0.002
	tMDl	Thalamus, mediodorsal lateral parvocellular	0.617	0.002
	tPuM	Thalamus, pulvinar medial	0.603	0.002
	tPuL	Thalamus, pulvinar lateral	0.538	0.009
	TPOsup	Temporal pole: superior temporal gyrus	0.495	0.015
	tVL	Thalamus, ventral lateral	0.490	0.016
	tLP	Thalamus, lateral posterior	0.481	0.018
	CERCRU2	Crus II of cerebellar hemisphere	0.461	0.021
	CER9	Lobule IX of cerebellar hemisphere	0.455	0.021
	CERCRU1	Crus I of cerebellar hemisphere	0.438	0.028
	CER7b	Lobule VIIB of cerebellar hemisphere	0.429	0.032
	INS	Insula	0.423	0.033
	tVPL	Thalamus, ventral posterolateral	0.405	0.039
	tAV	Thalamus, anteroventral nucleus	0.403	0.039
	tPuI	Thalamus, pulvinar inferior	0.399	0.041
	CER8	Lobule VIII of cerebellar hemisphere	0.395	0.042
	PHG	Thalamus, parahippocampal gyrus	0.385	0.046

## Discussion

Brain atrophy is a condition characterized by the loss of neurons and their connections, which can either be generalized or focal. Focal cerebral atrophy and the associated damage can affect specific areas of brain tissue, leading to corresponding functional impairments (34). Brain atrophy is also a crucial MRI marker in CSVD. Many studies reported an association between CSVD and brain atrophy, including global atrophy, corpus callosum atrophy, central atrophy (increased ventricular size and atrophy of the basal ganglia), mesencephalic atrophy, and hippocampal atrophy, as well as focal cortical thinning in brain regions connected to subcortical infarcts (3, 15, 16). Hippocampal and medial temporal lobe atrophy could contribute to cognitive impairment even in the absence of Alzheimer pathology (9, 35, 36). Alternatively, medial temporal lobe atrophy might associate with executive function impairment (37). A combined continuous measure of four imaging findings, including WMHs, lacunar, cerebral GM, and hippocampal volumes, had the greatest independent predictive value for both cognitive and functional outcome in older individuals with≤7 years follow-up (38). In additional that the CSVD-induced thalamic atrophy could mediate the effects of CSVD on slower walking speed in the elderly (8). Our study investigated the associations between distal arterial morphological features and brain atrophy indicators in CSVD. The results indicate that both distal artery length and density were positively correlated with GM fraction in CSVD patients, regardless of whether univariable or multivariable linear regression analyses were performed. In addition, distal artery length and density were also found to be negative associated with CSF fraction, although this relationship disappeared after adjusting for potential confounders. In further subgroup analysis, we also found that participants in the highest distal artery length tertile had significantly higher GM fraction and lower CSF fraction than participants in the lowest distal artery length levels. However, we did not find a significant association between average tortuosity and different brain structures. These results strongly suggested an intricate relationship between distal arterial features and different brain structures. To further investigate the relationship between distal arterial features and local changes in GMV, we divided GM into 170 regions using the AAL3. The study findings indicate a clear association between distal arterial morphologic features and regional GMV, as presented in Tables 6, 7. The corresponding GM regions images are depicted in Figures 4, 5. The parahippocampal gyrus (PHG), angular gyrus (ANG), anterior cingulate cortex (ACC), and insula (INS) are predominantly involved in emotion, cognition, and memory functions. Reduced GMV in these regions may lead to corresponding functional impairment. Furthermore, reductions in GMV in cerebellar structures are linked to symptoms of dizziness and unsteady gaits in patients with CSVD. A significant proportion of these GM nuclei are subcortical nuclear (caudate, putamen, pallidum, and thalamus), which play vital roles in motor control functions. Atrophy of these regions has been associated with worse walking performance in CSVD patients (8, 39, 40). The potential reason may be related with the following factors. The deep nuclei are vulnerable to direct ischemic or hemorrhagic damage associated with CSVD due to their surrounding small perforating arteries from the middle and posterior cerebral arteries (41, 42). Compared to larger arteries, the morphological characteristics of the distal arteries we studied may be more relevant to changes in these perforating arteries. Currently, there are limited studies on the association between cerebrovascular structure and CSVD. In a community-based cohort study, Zhang et al. (43) found that the detailed structural alterations in cerebral vessel radius, tortuosity, and density were correlated with WMHs. A cross-sectional study investigation conducted on young adults has revealed that there exists an inverse correlation between brain vessel density and WMHs lesion counts. This finding suggests that the vascular morphology of an individual might influence their white matter resilience and potential to withstand risk exposures (44). In another study, it was observed that an increase in carotid lumen diameter is associated with a higher prevalence of lacunar infarcts and WMHs volume (45). This could serve as a compensatory mechanism to counteract the increase in stiffness and thickness of the arterial wall, thereby maintaining arterial compliance in the normal values (46–48). All of aforementioned studies were restricted to the entire cerebral artery or the larger artery, while our investigation specifically targets the smaller artery. As widely recognized, CSVD encompasses a range of conditions characterized by pathological changes primarily affecting small blood vessels with a diameter ranging from 40-200 μm in the brain. Nonetheless, we were unable to quantitatively evaluate these vessels directly. Inadequate perfusion of small arteries may lead to decreased blood flow in the upstream arteries, resulting in morphological alterations in vascular features as observed on 3D-TOF MRA (25). These features are mainly derived from the distal arteries (A2, M2, P2 and more distal). In this study, as anticipated, we found that distal arterial morphologic features on 3D-TOF MRA are associated with total or regional brain volumes/atrophy of CSVD. This finding provides valuable information to clarify the relationship between vascular health indicators and brain atrophy, and offers new evidence for a possible vascular origin of brain atrophy.

The intracranial artery features observed on MRA should not be considered as an accurate representation of the true vascular morphology within the brain. Rather, they serve as proxies for various factors that affect the visibility and luminal signal of distal arteries (such as A2, M2, P2 and more distal) on MRA scans (21). These factors include, but are not limited to, brain blood flow, as well as wall thickening-induced luminal narrowing (20, 49, 50). Additionally, most non-contrast MRA techniques rely to some extent on blood flow for imaging purposes (17). A lower velocity and longer travel distance of blood flow spins result in a greater number of radiofrequency (RF) pulses experienced by the spins, leading to increased magnetization saturation and reduced visibility of arteries (49, 50). This may result in a decrease in calculated artery length and artery density, and consequently exhibit a lower mean artery tortuosity. To date, quantifying the morphologic features of intracranial distal arteries remain very complex and challenging. The iCafe has been developed and applied as a potent tool to tackle this issue. As per the research conducted by Gould et al. (20), the length of visible intracranial arteries measured by iCafe based on TOF MRA can be employed as a promising surrogate imaging marker for brain blood flow. In another study, it was discovered that the length of intracranial arteries and the number of branches observed on TOF MRA were linked to Montreal Cognitive Assessment (MoCA) scores. These findings were not influenced by carotid stenosis, age, systolic blood pressure, use of anti-hypertensive drugs, or cerebral blood flow (CBF), indicating that they could offer supplementary insights into cerebrovascular health beyond conventional blood flow measurements (21). As commonly acknowledged, advanced age and hypertension are the primary risk factors for CSVD. Through the utilization of the iCafe, Chen et al. (18) conducted a study that demonstrated a negative correlation between older age and the visibility of distal artery vascularity, as evidenced by quantitative assessments of the number of branches, average artery order, and average tortuosity in a substantial sample of older adults. Additionally, in a community-based study, high blood pressure (BP) was found to be linked to decreased vessel density and branch numbers, which could signify inadequate perfusion of small arteries, thereby contributing to the development of small vessel disease (43). These studies suggested that cerebrovascular morphological indicators closely related to advanced age and hypertension were appropriate for evaluating CSVD. Our study highlights the close association between distal arterial morphological features and generalized or focal brain atrophy indicators in CSVD, which has yet to be reported. As previously suggested, our study updated the method for evaluating the morphological characteristics of intracranial arteries in CSVD, contributing to the limited research on the link between vascular health indicators and brain atrophy, offering new evidence for a possible vascular origin of brain atrophy. Given its non-invasive and clinically valuable nature, the morphological features of intracranial distal arteries based on 3D TOF-MRA will be promising indicators for widespread application in CSVD.

There are also certain limitations to this study. Firstly, the sample size is relatively small, which may lead to insufficient power for observing some potential links between the morphological characteristics of intracranial distal arteries and measures of brain atrophy. Secondly, the TOF sequence did not encompass over the whole brain, and some very distal branches, especially that of the ACA, were not measurable. This could have potentially affected the accuracy involving the vessel length, volume and tortuosity. Thirdly, current vessel tracing with the tool iCafe is still time consuming (approximately 2 h per case) due to the need for manual corrections, which could limit its clinical utility. Fourthly, the inclusion of participants with undetected hereditary cerebrovascular disease may result in differences in cerebrovascular morphology compared to other participants. Fifthly, while no other clinical diagnosis of neurological disorders were identified among all participants, subclinical pathologies related to neurodegenerative diseases cannot be excluded. The relationship between these subclinical pathologies and cerebrovascular morphologic features is not clearly identified. Sixthly, we did not adjust our analyses for other MRI markers of CSVD, including cerebral microbleeds, lacunes, or subcortical infarct.

## Conclusion

The morphologic features of intracranial distal arteries, including artery length, density and average tortuosity, measured from 3D-TOF MRA, are associated with generalized or focal brain atrophy indexes. Our study updated the method for evaluating the morphological characteristics of intracranial arteries in CSVD, providing valuable information to clarify the relationship between vascular health indicators and brain atrophy, and offering new evidence for a possible vascular origin of brain atrophy.

## Data availability statement

The original contributions presented in the study are included in the article/supplementary material, further inquiries can be directed to the corresponding author.

## Ethics statement

The studies involving human participants were reviewed and approved by Ethics committee of Jincheng People’s Hospital, China. The patients/participants provided their written informed consent to participate in this study.

## Author contributions

JT put forward research ideas, revised the manuscript, and provided expert opinion. HL and CL took the responsibility of communicating with the patient’s family and obtaining the authorization in this study. CL, HL, and DZ collected the clinical characteristics data. LJ and LX provided statistical support, reviewed the manuscript, and provided expert opinion. HC and HL collected and analyzed the brain MRI data and also conducted the statistical analysis. HC designed and conceptualized the study, analyzed the data, wrote, and revised and edited the manuscript. All authors contributed to the article and approved the submitted version.

## Conflict of interest

The authors declare that the research was conducted in the absence of any commercial or financial relationships that could be construed as a potential conflict of interest.

## Publisher’s note

All claims expressed in this article are solely those of the authors and do not necessarily represent those of their affiliated organizations, or those of the publisher, the editors and the reviewers. Any product that may be evaluated in this article, or claim that may be made by its manufacturer, is not guaranteed or endorsed by the publisher.

## Footnotes

^1^ http://www.fil.ion.ucl.ac.uk

^2^ http://www.neuro.uni-jena.de/cat12/CAT12-Manual.pdf

^3^ www.statistical-modelling.de/lst.html
